# POstLAparoscopic Reduction of pain By combining intraperitoneal normal salinE And the pulmonary Recruitment maneuver (POLAR BEAR trial). RCT to estimate reduction in pain after laparoscopic surgery when using a combination therapy of intraperitoneal normal saline and the pulmonary recruitment maneuver

**DOI:** 10.1186/s12905-017-0397-8

**Published:** 2017-06-13

**Authors:** J. E. W. van Dijk, S. J. Dedden, P. M. A. J. Geomini, P. Meijer, N. van Hanegem, M. Y. Bongers

**Affiliations:** 1 Department of Obstetrics and Gynecology, MUMC+, GROW - School for Oncology and Developmental Biology, PO Box 5800, 6202 AZ Maastricht, the Netherlands; 2 Department of Obstetrics and Gynecology, Máxima Medical Center, PO Box 777, 5500 MB Veldhoven, the Netherlands; 3 Department of Anesthesiology, Máxima Medical Center, PO Box 777, 5500 MB Veldhoven, the Netherlands

**Keywords:** Postlaparoscopic pain, Shoulder pain, Pulmonary recruitment maneuver, Intraperitoneal saline

## Abstract

**Background:**

Shoulder pain and pain in the upper abdomen are common complaints after laparoscopy, sometimes surpassing the pain at incision sites. The incidence of shoulder pain ranges from 35 to 80%. Post-laparoscopic pain is caused by retention of carbon dioxide in the abdomen, which irritates the phrenic nerve and diaphragm, causing referred pain in the shoulder and in the upper abdomen. A promising strategy to reduce this post-laparoscopic pain is the pulmonary recruitment maneuver, which indirectly increases intraperitoneal pressure and thereby facilitates removal of residual carbon dioxide. An alternative strategy is the infusion of intraperitoneal normal saline. With normal saline infusion, carbon dioxide rises and escapes through the port sites. In addition, normal saline offers a physiologic buffer system to dissolve excess carbon dioxide.

**Methods/Design:**

This multicenter randomized controlled trial is conducted in two teaching hospitals in the Netherlands. Women between 18 and 65 years of age, with an ASA classification of I-II who are scheduled to undergo an elective laparoscopic procedure with benign gynecologic indication can participate. Following informed consent, participants are randomly allocated into two groups at the end of the surgical procedure. In the intervention group, the upper abdomen is filled with normal saline infusion with the patient in the Trendelenburg position. Then the anesthesiologist performs a standardized pulmonary recruitment maneuver with a pressure of 40 cm H_2_O. The trocar sleeve valves will be left open, so carbon dioxide can escape the abdominal cavity. With the patient in a neutral position the instruments are removed from the abdomen. In the control group, carbon dioxide is removed from the abdominal cavity at the end of surgery, with gentle abdominal pressure and passive exsufflation through the port sites, with open sleeve valves.

The primary outcomes are the incidence and intensity of post-laparoscopic pain in the shoulder, upper abdomen and at the operation sites, at 8, 24 and 48 h after surgery. Secondary outcomes are postoperative use of analgesics, nausea, vomiting and pulmonary complications.

**Discussion:**

This study may reduce post-laparoscopic pain in women undergoing laparoscopy.

**Trial registration:**

Dutch trial register, number NTR4812.

## Background

Laparoscopic surgery has become a common surgical practice. It is associated with a shorter hospital stay, earlier return to daily activities and work and improved cosmetic results compared with open procedures [1]. Despite these advantages, many patients suffer from pain in the shoulder and upper abdomen after laparoscopy. The incidence of shoulder pain ranges from 35 to 80% [2–8].

Sometimes this post-laparoscopic shoulder pain surpasses the pain at the incision site [6]. Nowadays, due to early discharge, post-laparoscopic pain will not be noticed by the physicians and therefore not treated properly.

The cause of post-laparoscopic pain is not fully understood. Several factors may contribute to this pain. Rapid distention of the peritoneum may result in traumatic traction on blood vessels and nerves with inflammatory mediator release and phrenic nerve neuropraxia [9–11]. Therefore pressure peaks and prolonged insufflations should be avoided [5, 10]. In addition, the phrenic nerve may be damaged by the acidotic and cooling effect of insufflated carbon dioxide. This may result in an irritative effect on the peritoneum and diaphragm [10, 12–15]. Furthermore, post-laparoscopic pain is thought to be caused by retention of carbon dioxide in the abdomen, which irritates the phrenic nerve and diaphragm and causes referred pain in the shoulder and pain in the upper abdomen [2, 9, 10, 16]. A significant correlation between the amount of residual pneumoperitoneum and pain scores has been reported [2, 9, 16]. It is not clear whether the length of the surgical procedure has an effect on the intensity or incidence of post-laparoscopic pain [17, 18].

Two promising strategies to reduce post-laparoscopic pain after gynecological surgery are mentioned in the literature [1, 19, 20]. The first strategy, the pulmonary recruitment maneuver, is designed to open alveoli, which increases intrapulmonary pressure. As a result, intraperitoneal pressure will rise and facilitate the removal of residual carbon dioxide from the abdomen [7, 19–22]. A second strategy involves the use of intraperitoneal normal saline. By filling the abdomen with warmed normal saline, carbon dioxide rises and escapes through the port sites [23]. In addition, normal saline is thought to offer a physiologic buffer system to dissolve excess carbon dioxide [19]. A combination of the two techniques described above has been shown to reduce post-laparoscopic pain in Asian women with a mean body weight < 60 kg [20].

We propose a randomized controlled trial to study the effect of a combination of intraperitoneal saline and the pulmonary recruitment maneuver on the incidence and intensity of post-laparoscopic pain in Dutch women.

## Methods/design

### Objective

The aim of this study is to assess the incidence and intensity of post-laparoscopic pain in the shoulder, upper abdomen and at the operating site 8, 24 and 48 h after elective gynecologic laparoscopic surgery with benign indication. This study will also evaluate postoperative use of analgesics, occurrence of nausea and vomiting and pulmonary complications.

### Trial design

This study is a randomized controlled trial and will be performed at the Maastricht University Medical Center, a university hospital in the Netherlands, and at Máxima Medical Center Veldhoven, a tertiary teaching hospital in the Netherlands.

The study is conducted according to the principles of the Declaration of Helsinki and in accordance with the Medical Research Involving Human Subjects Act (WMO) and has been approved by the ethics committee of Máxima Medical Center (METC no 1445, CCMO no NL50655.015.14). The protocol is registered in the Dutch Trial register, number NTR4812.

The trial will be conducted without any funding sources.

### Eligibility criteria

Women between 18 and 65 years of age with an American Society of Anesthesiologists physical status classification of I-II (ASA classification) who are scheduled to undergo an elective laparoscopic procedure, with a benign gynecologic indication, can participate in the trial.

The first exclusion criterion is daily use of analgesics because postoperative use of analgesics is a secondary outcome. The next exclusion criterion is allergy/intolerance to nonsteroidal anti-inflammatory drugs (NSAIDs), as these are the standard postoperative analgesics used. Emphysema and chronic obstructive pulmonary disease (COPD) are also exclusion criteria for the pulmonary recruitment maneuver is used. A midline laparotomy in the medical history is another exclusion criterion, as this is often a reason to perform an open laparoscopy and it increases the risk of conversion to laparotomy. The last exclusion criterion is poor understanding of the Dutch language. This was deemed necessary because we make use of questionnaires written in Dutch.

### Patient recruitment, randomization and collection of data

Eligible patients are identified by the gynecologist or the resident in one of the two participating hospitals. Women eligible for the trial will be counseled by experienced research nurses or researchers. They will be informed about the aims, methods, reasonably anticipated benefits and potential hazards of the study. After a week patients will be asked if they want to participate and informed consent will be collected. By giving written informed consent, patients agree to participate and agree that their anonymous data may be used for publication.

Randomization will be performed by using sealed envelopes and will take place just before the end of the surgery.

The study is blinded for patients, but not for the gynecologist, as the gynecologist is the one who will leave normal saline intraperitoneal when necessary. Because this study is a multicenter trial, randomization will be stratified by hospital to get an equal distribution of patients between the two groups (control and intervention) per hospital. For randomization we used an Excel sheet that randomly assigned 200 patients (two lists of 100 patients, one for each hospital) to either the intervention or control group. Data handling will be done anonymously and the date code is only available to the local investigator. The baseline data and follow-up data collection at both hospitals is the responsibility of the two local investigators. Data will be stored in a sealed, anonymous files.

The dataset supporting the conclusions of this article will be available upon request. In accordance with the guidelines of the Dutch Federation of University Medical Centers (NFU), the data will be kept for 15 years.

### Interventions

In the intervention group, the patient will be placed in the Trendelenburg position of 30 degrees at the end of the laparoscopic procedure. An intraperitoneal saline infusion of 15–20 ml/kg will be administered evenly and bilaterally by the operating gynecologist. The anesthesiologist will perform the pulmonary recruitment maneuver after the saline infusion. Five pulmonary insufflations with a pressure of 40 cm H_2_O (pulmonary recruitment maneuver) will be given. The fifth positive pressure inflation will be held for 5 sec﻿. The trocar sleeve valves will be left fully open during this procedure, so the carbon dioxide can escape the abdominal cavity. Hereafter the patient is placed in a neutral position and the instruments are removed from the abdomen.

In the control group, the carbon dioxide is removed from the abdominal cavity at the end of surgery, with gentle abdominal pressure and passive exsufflation through the port sites with the sleeve valves open.

### Measurement and follow-up

Primary outcomes are the incidence and intensity of post-laparoscopic pain in shoulder, upper abdomen and at the operating site at 8, 24 and 48 h after surgery, which are measured using the Visual Analogue Scale (VAS). Secondary outcomes are postoperative use of analgesics, nausea, vomiting and pulmonary complications. We will record patient characteristics (age, height, weight, body mass index (BMI)) and operative details (type of surgery, ASA-classification, duration of surgery, estimated blood loss, total volume of gas used, amount of normal saline left intraperitoneal, and postoperative hospital stay).

### Statistical analysis

#### Sample size

The two primary outcomes of our study are the incidence of pain and the intensity of pain using the VAS score. In accordance with Tsai et al. [20], we have measured pain intensity using the preliminary data of the 24-h and 48-h pain scores. Post-laparoscopic pain is most common and evident in the first 48 h after surgery, after that the pain declines. That is why we have chosen to look at the 48-h pain score. Furthermore, we have assumed a reduction in the incidence of pain from 80% to 50% using this combination treatment compared to standard treatment [7, 24]. A dropout rate of 20% is anticipated because of loss to follow-up or incomplete questionnaires. We use Fisher’s exact test to test for differences in the proportion. To do so, we need to include at least 126 patients (63 women in each study arm) to obtain a power of 85% when testing with an alpha of 5%.

#### Data analysis

Data will be analyzed according to the intention–to-treat principle. Statistical analysis will be performed using the software Statistical Package for the Social Sciences 22.

Categorical variables will be described as frequencies and percentages. Continuous variables will be described in terms of means with standard deviation if normally distributed, and otherwise as a median with an interquartile range (IQR). Significance will be tested two sided with an α-level of 0.05. Patients will be classified as either having pain or not having pain using the questionnaires the patients fill in after surgery. To compare the incidence of pain between the intervention and control groups, the Pearson’s Chi-squared statistic will be used. For those patients experiencing pain, the average pain scores between groups will be compared. T-tests for pain scores will be used when the scores are normally distributed.

## Results

After randomization we included 56 patients in the control group and 71 patients in the intervention group. The unequal randomization was caused by using the incorrect method, as we should have used a block randomization method. To correct the unequal distribution, we requested an amendment which was approved by the ethics committee. We wanted to include 200 women, resulting in 100 women in each arm. To reach this number of inclusions, we had to include an additional 29 women in the intervention group and an additional 44 women in the control group.

## Discussion

In light of the increasing number of laparoscopic interventions [25], the short postoperative stay of patients and the high incidence of post-laparoscopic shoulder and upper abdominal pain [2–8], interventions to decrease post-laparoscopic pain are important and necessary in order to deliver good medical care. The combination of intraperitoneal saline infusion and the pulmonary recruitment maneuver has shown to decrease post-laparoscopic pain in Asian women with a mean body weight < 60 kg [20]. This study will be performed in the Netherlands in a population of West-European women with a higher average BMI, who will undergo a gynecologic laparoscopy for benign indication. The results will therefore be applicable for a large population of patients undergoing elective benign laparoscopy.

## Abbreviations

ASA classification: American society of anesthesiologists
POLAR BEAR trial: Postlaparoscopic reduction of pain by combining intraperitoneal normal saline and the pulmonary recruitment maneuver
RCT: Randomized controlled trial
VAS score: Visual analogue scale
